# Barriers to Research Publication for Undergraduate Medical Students in Makkah, Saudi Arabia: A Cross-Sectional Study

**DOI:** 10.7759/cureus.69502

**Published:** 2024-09-16

**Authors:** Sari A Alrehaili, Abdullah H Binmulaih, Mohammed M Alshomrani, Abdullah S Alnefaie, Ahmad O Bazarra, Mohammad S Dairi

**Affiliations:** 1 College of Medicine, Umm Al-Qura University, Makkah, SAU; 2 Internal Medicine, Umm Al-Qura University, Makkah, SAU

**Keywords:** academic teaching, article, barriers, cross-sectional, medical research, publication, research curriculum, saudi arabia, students, undergraduate

## Abstract

Background

Scientific research is an approach to acquiring information to enhance the quality of the world. The aim of this study was to identify the obstacles among undergraduate medical students to publish their study papers.

Materials and methods

This was a web-based descriptive cross-sectional study that used an online survey to collect data from medical students of Umm Al-Qura University in Makkah, Saudi Arabia. The minimum sample size required for the study calculated by the Raosoft website was 323. Statistical analysis was conducted with IBM SPSS Statistics for Windows, Version 25 (Released 2017; IBM Corp., Armonk, New York, United States).

Results

A total of 337 medical students were included in the study. The frequencies of male and female students were relatively comparable, with a marginally greater proportion of male students (177, 52.5%). The majority of students (294, 87.2%) had participated in at least one research project. Notably, 159 (47.2%) students had engaged in more than three research projects, whereas 42 (12.4%) had experience with only one previous research project. A notable peak in publication rate was observed for the year 2023, indicating the highest rate within the study period. The most frequently encountered obstacle in research publication was poor academic teaching, as reported by 133 (39.5%) students, followed by lack of active research groups (130, 38.6%), lack of time (122, 36.2%), and lack of supervisors in their preferred specialty (91, 27%). Associations were found between students’ participation in research projects and various demographic characteristics. Among medical students who had previously participated in a research group, 174 (51.7%) were female, and 163 (48.3%) were male (p=0.001). In terms of academic year, most students who participated in a research project were in their sixth year (143, 42.5%), followed by medical interns (81, 24.1%) and fourth-year medical students (54, 16%), with these differences being statistically significant (p=0.001). Finally, the medical students’ grade point average showed no statistically significant association with participation in a research project.

Conclusion

To boost publication rates and research experience of graduating medical students, it is important to articulate the barriers that prevent them from publishing their research. Educators and supervisors of medical research should strongly consider developing a framework that addresses current and future obstacles.

## Introduction

Scientific research is a methodical approach to acquiring information and utilizing curiosity to enhance the quality of the world. For example, research is crucial for the development of medicine, as it is required in shaping evidence-based medical practice [1-5]. A 2010 Peruvian study on medical students at Cayetano Heredia University revealed that, out of 482 students' scientific papers, only 85 (17.6%) research papers were accepted to be published; however, other papers were rejected [6]. A 2011 British study on medical students at Manchester University revealed that, out of 515 students, only 72 (14%) submitted their scientific papers for publication due to a lack of time and opportunity [2]. In 2012, a local study of 249 medical interns from King Abdulaziz University showed that only eight (3.2%) submitted and published their research due to a lack of training in the field of research [7]. In 2023, a local study of 162 undergraduate medical students in Riyadh, Saudi Arabia, revealed numerous obstacles faced when publishing scientific papers, including unsupportive supervisor (63, 38.9%), lack of time (50, 30.9%), insufficient sample size (41, 25.3%), inability to reconcile research and studying (37, 22.8%), poor methodology (31, 19.1%), advanced topic/field of research (29, 17.9%), limited access to databases (16, 9.9%), lack of funding (15, 9.3%), and language barriers (7, 4.3%) [8]. According to the scientific research unit of the Faculty of Medicine at Umm Al-Qura University (UQU), out of 235 medical students, only 48 (20.43%) published a scientific paper by their fifth or sixth years. From all the data that were obtained, no clear determinant was found to determine the cause of the low publication rate among students in Saudi Arabia.

The medicine program at UQU provides courses throughout the academic years for students to learn and publish papers with the help and supervision of university doctors. Our primary objectives in this study were to assess and determine the obstacles and barriers faced by undergraduate medical students in UQU when publishing scientific papers, identify any determinants, and present solutions. Our secondary objectives were to determine the challenges and opportunities faced by undergraduate medical students in UQU when conducting research projects.

## Materials and methods

Study design, setting, and population

This was a web-based descriptive cross-sectional study. An online survey was used to collect data from undergraduate medical students attending UQU in Makkah, Saudi Arabia.

Sample size and study population

According to the scientific research unit of the Faculty of Medicine at UQU, there were approximately 2000 students studying at UQU at the time of this study. Therefore, based on the Raosoft sample size calculator (Raosoft Inc., Seattle, USA) while assuming a 95% confidence level and 5% margin of error, the minimum recommended sample size was calculated to be 323 individuals.

Study period and duration

The study lasted approximately four months, from March 2024 to July 2024, including the entire process of writing the proposal to submit the article to the journal including data collection process that was held in the period from April 2024 to May 2024.

Survey design and pilot study

The questionnaire used in this study was validated by expert opinions and piloting study. The questionnaire’s test-retest reliability was evaluated by administering it multiple times during the pilot phase and observing consistent responses. The sample size is large, which contributes to the reliability of Cronbach's Alpha. Through this iterative process, we arrived at an optimized form of the questionnaire.

Data collection

An online survey was developed using Google Forms so that respondents could conveniently complete the questionnaire. The survey comprised two sections. In section 1, the respondents provided consent to participate in the study and sociodemographic characteristics. Section 2 comprised questions about students’ published or unpublished studies, study type, topic area, and barriers to publication. The questionnaire can be found in the appendix of this paper.

Statistical analysis 

Statistical analysis of the current study was conducted using IBM SPSS Statistics for Windows, Version 25 (Released 2017; IBM Corp., Armonk, New York, United States). Frequencies and percentages were used to express categorical variables. Items with multiple responses were analyzed using the multiple-response analysis technique. Factors associated with students’ participation in research projects were assessed using Pearson’s chi-squared test or Fisher’s exact test whenever applicable. A p-value of <0.05 indicated statistical significance, and the confidence interval (CI) was set at 95% for all analyses used in the study. We controlled for potential confounding variables such as individual motivation, prior research experience, and access to resources by ensuring homogeneity in these factors across our study groups. Moreover, we checked all the p-values for these variables, and none were found to be significant, indicating that these factors did not influence our results.

Ethical approval and ethical considerations

The study received ethical approval from the Research Ethical Committee of UQU on April 29, 2024 (approval no. HAPO-02-K-012-2024-04-2119). No study activities began until approval was obtained. Survey responses were collected in an anonymous fashion.

## Results

Demographic characteristics

A total of 337 medical students were included in the study. Table 1 presents the demographic characteristics of the participants. Regarding gender distribution, the frequencies of male and female students were relatively comparable, with a marginally greater proportion of male students (52.5% (177 students)). Most of the participants were in their sixth academic year (131, 38.9%), followed by medical interns (71, 21.1%), and fourth-year students (65, 19.3%). More than half of students recorded a grade point average (GPA) between 3.50 and 4.0 with 73.3% (247 students).

**Table 1:** Demographic data of medical students at Umm Al-Qura University during the academic year 2023-2024 GPA: grade point average

Characteristic		Frequency	Percentage
Gender	Male	177	52.5%
	Female	160	47.5%
Academic year	2nd year in medical school	6	1.8%
	3rd year in medical school	2	0.6%
	4th year in medical school	65	19.3%
	5th year in medical school	62	18.4%
	6th year in medical school	131	38.9%
	Medical intern	71	21.1%
Current GPA (out of 4)	3.50-4	247	73.3%
	3.00-3.49	66	19.6%
	2.50-2.99	23	6.8%
	<2.50	1	0.3%

Previous research experiences of medical students

Table 2 outlines the previous research experiences of medical students at UQU. The majority of students had participated in at least one research project, with 294 students (87.2%) indicating participation. A notable portion of students had engaged in more than three research projects (159, 47.2%), whereas 42 students (12.4%) described experience in only one previous research project. Regarding research publication frequency, nearly half of the participants had not yet published any research project (164, 48.7%). Meanwhile, 59 students (17.5%) contributed to the publication of one research project. Fewer students had higher publication frequencies, with 37 students (11.0%) having two research projects published, and even fewer, 25 students (7.4%), with three research publications.

**Table 2:** Previous research experiences of medical students at Umm Al-Qura University, 2023-2024

Characteristic		Frequency	Percentage
Previous participation in a research project	Participated	294	87.2%
	Did not participate	43	12.8%
Frequency of research participation	One research project	42	12.4%
	Two research projects	43	12.8%
	Three research projects	50	14.8%
	More than three research projects	159	47.2%
	None	43	12.8%
Frequency of research publication	One research project	59	17.5%
	Two research projects	37	11.0%
	Three research projects	25	7.4%
	More than three research projects	52	15.4%
	None	164	48.7%

**Figure 1:**
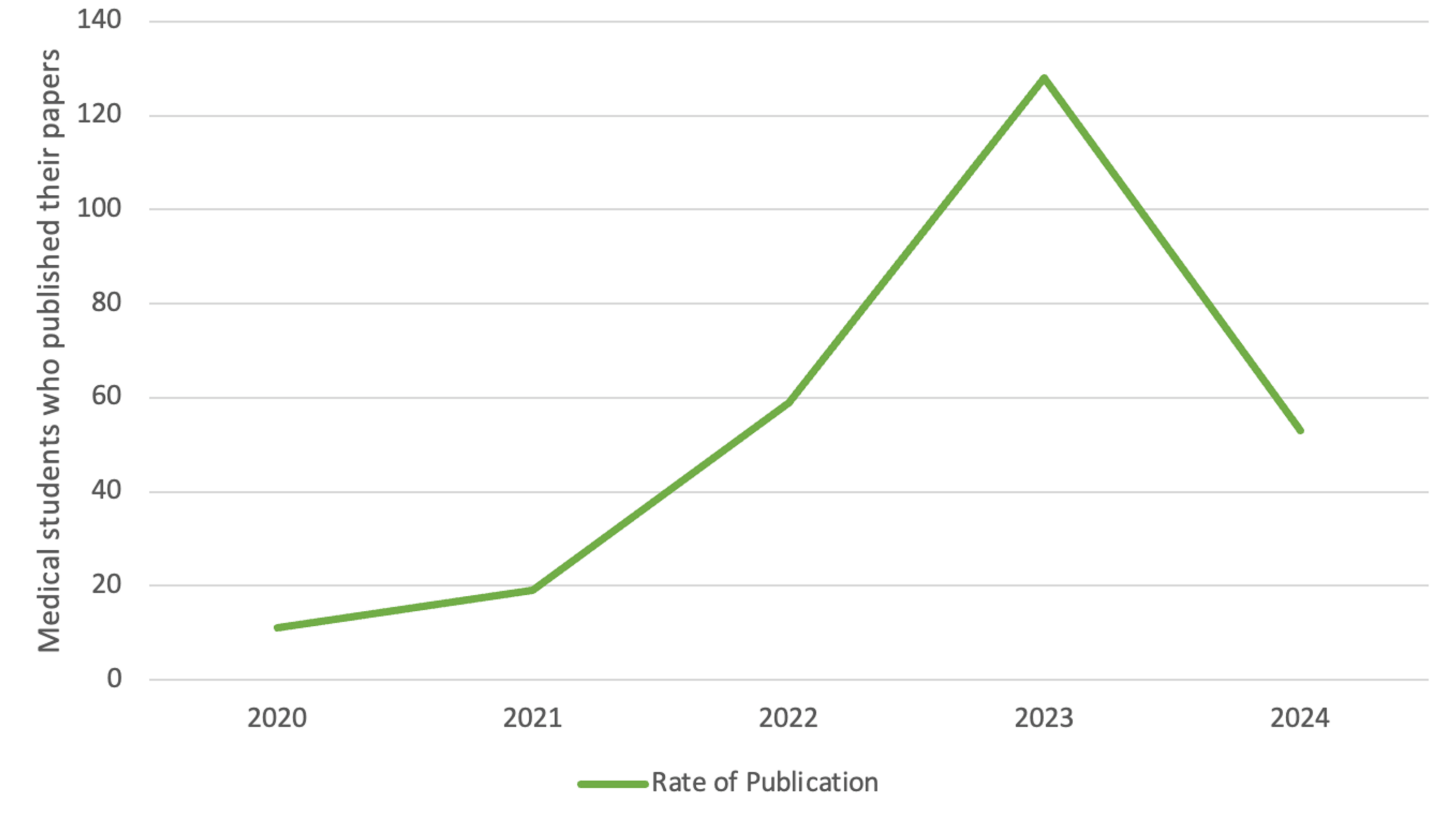
The rate of research publication among medical students at Umm Al-Qura University who had previously published a research project

**Figure 2:**
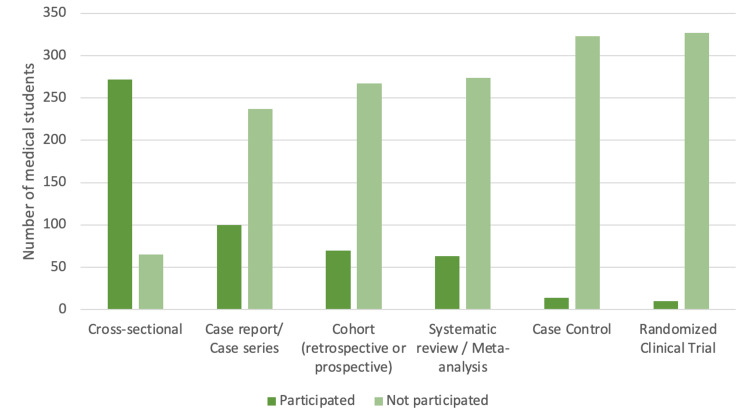
Types of study design participated in/published by medical students at Umm Al-Qura University, 2023-2024

Figure 1 illustrates the annual trend in research publication rates among the students who had engaged in research. There was a notable peak observed in 2023, indicating the highest publication rate within the depicted time frame. Figure 2 summarizes the types of study designs in which the medical students engaged. Cross-sectional studies had the most substantial participation from students (272 projects), followed by case reports/case series (100 projects) and cohort studies (70 projects).

Medical students’ obstacles in research publication

**Figure 3:**
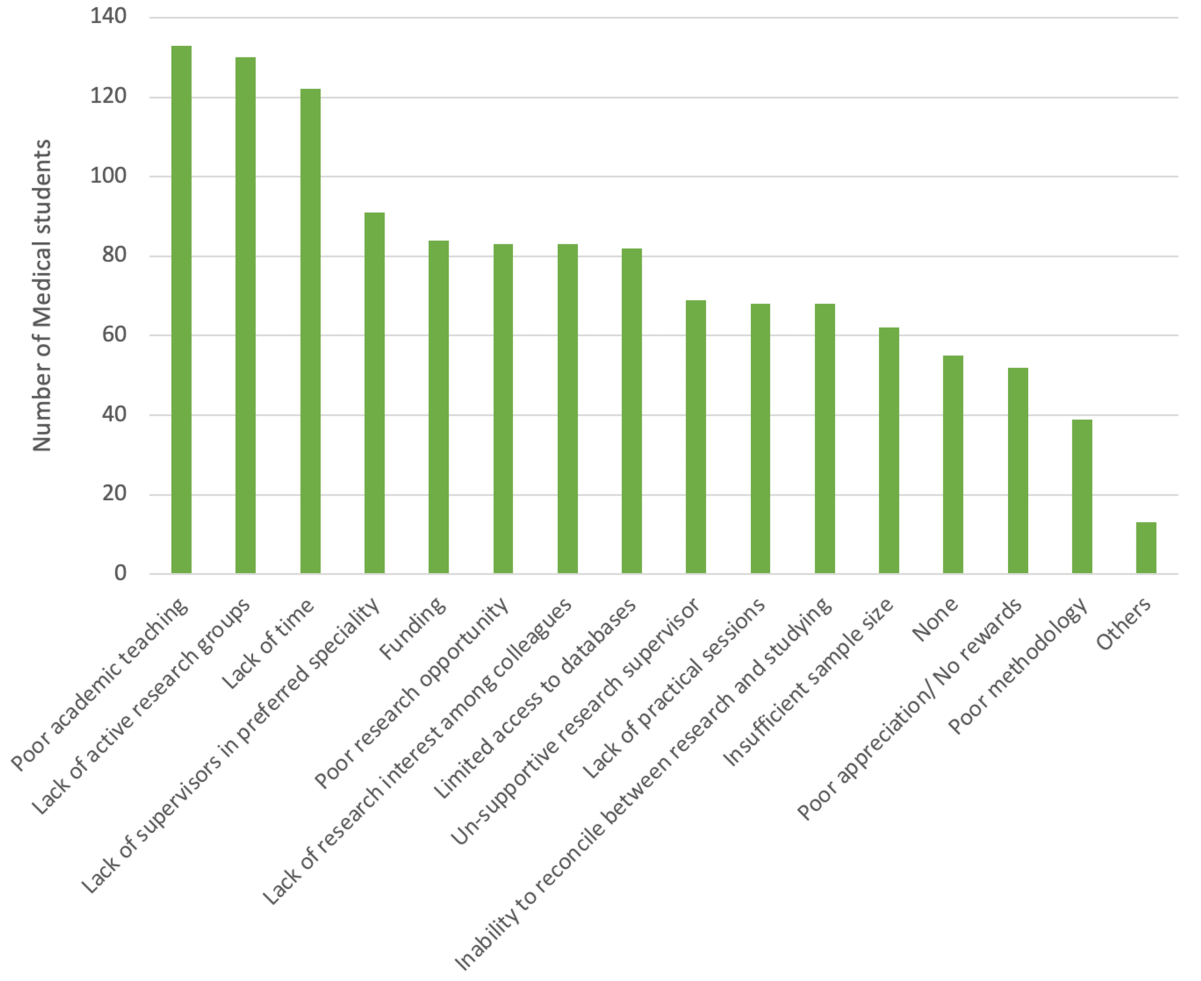
Medical students’ obstacles in research publication at Umm Al-Qura University, 2023-2024

According to the participants’ responses, the most commonly encountered obstacle in research publication was poor academic teaching (133, 39.5%), followed by a lack of active research groups (130, 38.6%), a lack of time (122, 36.2%), and a lack of supervisors in their preferred specialty (91, 27%). Figure 3 outlines the obstacles and barriers reported by the medical students.

Associations between research participation and demographic factors

Table 3 summarizes the associations between students’ participation in research projects and demographical factors. Among medical students who had previously participated in a research project, 174 participants (51.7%) were female and 163 participants (48.3%) were male, which was a statistically significant difference (p=0.001). In terms of the academic year, most students who participated in a research project were in their sixth year (143, 42.5%), followed by medical interns (81, 24.1%) and fourth-year medical students (54, 16.0%). These findings recorded a p-value of 0.001, indicating statistical significance. No statistically significant association between GPA and participation in a research project was observed.

**Table 3:** The association between students’ participation in research projects and demographical factors GPA: grade point average ^*^ represents statistical significance (p<0.05); ^§^ Pearson Chi-square test and likelihood ratio were used to calculate the p-value depending on the number of cells expected to count less than 5.

Characteristic	Participation in a research project		Chi-square value	p-value^§^
	Participated	Not participated		
Gender				
Male	142 (48.3%)	35 (81.4%)	16.478	0.001^*^
Female	152 (51.7%)	8 (18.6%)		
Academic year				
2nd year in medical school	3 (1.0%)	3 (7.0%)	52.778	0.001^*^
3rd year in medical school	2 (0.7%)	0 (0.0%)		
4th year in medical school	47 (16.0%)	18 (41.9%)		
5th year in medical school	46 (15.6%)	16 (37.2%)		
6th year in medical school	125 (42.5%)	6 (14.0%)		
Medical Intern	71 (24.1%)	0 (0.0%)		
Current GPA (out of 4)				
3.50-4	222 (75.5%)	25 (58.1%)	6.434	0.069
3.00-3.49	54 (18.4%)	12 (27.9%)		
2.50-2.99	17 (5.8%)	6 (14.0%)		
<2.50	1 (0.3%)	0 (0.0%)		

## Discussion

Determining the barriers that keep students from publishing their work is essential to scientific progress and the primary objective of the current study. Few research studies address and acknowledge these obstacles in undergraduate medical curricula, especially in Saudi Arabian universities [7-9]. The findings of this study indicate that only half of the students at UQU published research papers, with higher publication rates compared to other similar studies that found low publication rates among undergraduate medical students [6,10,11]. However, in a German study, 28% of the participants had publications [12]. In a US study, 41% of the student participants had published research papers, similar to our study [13]. Numerous factors are responsible for the variations in publishing rates over time and between colleges. The most common barrier in our study was poor academic teaching followed by a lack of active research groups. As the majority of supervisors work as full-time teachers at universities and physicians simultaneously, research projects are an additional obligation for them. Moreover, this barrier appears to be more prevalent than predicted in previous studies. A survey conducted by Australian medical researchers revealed that research infrastructure support is critical to research productivity [14]. As expected, a major portion of the issues that our students encountered were related to time constraints and their incapacity to balance their studies and research. Furthermore, funding, lack of research opportunities, lack of research interest among colleagues, limited access to databases, and unsupportive research supervisors were also reported as obstacles.

In a study conducted at King Saudi bin Abdulaziz University for Health Sciences, poor English was reported as an obstacle to research publication, which was not measured in our study [4]. Insufficient sample size was another obstacle facing medical students at UQU due to students’ lack of interest in filling out research questionnaires. Another interesting barrier medical students faced in our study was limited access to databases, which is crucial to conducting high-quality studies. The students study the research curriculum in multiple lectures over the years. However, these lectures are limited to theoretical sessions, which leads to another of the barriers reported as an obstacle in our study, a lack of practical sessions. Furthermore, UQU has mandatory research courses for undergraduate medical students at all academic levels. Our results indicate that students carry negative attitudes toward medical research curricula due to multiple factors, such as spaced research lectures and lack of practical sessions.

In terms of demographic factors associated with research participation, our results showed a statistically significant sex-based difference with higher female participation. Furthermore, most participants were sixth-year medical students, followed by medical interns (p=0.001). There was no statistically significant association between GPA and research publication. Although the majority of students who participated in our study engaged in one research project, nearly half of the participants had not yet published any research. These findings agreed with those of previous studies on publication rates among medical students [6,11]. Students must overcome these challenges by relying on their willingness to embrace suitable solutions, such as improving time management skills, collaborating with cooperative mentors, and strengthening their essential biostatistical abilities to plan proper methodology [15-17].

This study had many strengths, as it had a large number of participants, it covered medical students throughout all academic years, and it attempted to identify possible obstacles to publication practices. The authors highly recommend giving more lectures and practical sessions to enhance students’ ability in scientific research. Moreover, holding more research medical conferences could enhance students’ ability to conduct medical research.

Although our study findings may be beneficial for undergraduate medical students from UQU, their usefulness for students of other universities may be limited. As each institution has a unique curriculum and approach to research, our findings cannot be applied to all research programs. Future studies should investigate gaps in the rate of publication and difficulties reported by students according to their academic year as well as other student demographic variables. Furthermore, due to the COVID-19 pandemic, the research course at UQU has been taught online exclusively for two years. It would be fascinating to investigate the challenges that students have and find possible solutions to improve the quality of undergraduate medical students research papers.

## Conclusions

Medical students have a poor understanding of how to write papers and abstracts, as well as publication methods, despite the fact that such abilities are employed as postgraduate criteria when selecting candidates for positions. Medical students encounter several obstacles that hinder their chances of publishing papers, including working with an unsupportive supervisor, insufficient time, and sample size limitations. To overcome these barriers and improve students’ research process and quality, further studies are needed to provide a clearer image and practical ideas. This process may be enhanced by making research opportunities more available and providing encouragement and advice from mentors.

## Appendices

**Table 4:** The questionnaire of the study

Consent for participation	By answering this survey you are participating in research entitled "Barriers to Research Publication for Undergraduate Medical Students in Makkah, Saudi Arabia: A Cross-Sectional Study." Data will be used for medical research while protecting the privacy of participants’ personal data.	Agree to participate in the study
		Disagree
Section 1: Sociodemographics	Are you studying at Umm Al-Qura University?	Yes
		No
	What is your gender?	Male
		Female
	What is your current academic year?	2nd year medical student
		3rd year medical student
		4th year medical student
		5th year medical student
		6th year medical student
		Medical intern
	What is your current GPA? (Out of 4)	3.5–4
		3–3.49
		2.5–2.99
		<2.5
Section 2: Main questionnaire	Have you participated in any previous research?	Yes
		No
	How many research projects did you participate in?	None
		1
		2
		3
		>3
	How many research papers did you publish?	None
		1
		2
		3
		>3
	When did you publish your research?	Not yet
		2020
		2021
		2022
		2023
		2024
	What is the research study design?	Case report/series
		Case control
		Cross-sectional
		Cohort
		Randomized clinical trial
		Systematic review/meta-analysis
	What were the obstacles that you faced in publishing your research? (choose as many as applicable)	Funding
		Lack of time
		Poor academic teaching
		Advanced topic
		Poor methodology
		Poor research opportunity
		Lack of supervisors in your preferred speciality
		Lack of active research groups
		Unsupportive research supervisor
		Poor appreciation/ No rewards
		Limited access to databases
		Insufficient sample size
		Inability to reconcile between research and studying
		Lack of research interest among your colleagues
		Lack of practical sessions
		None
		Others
